# *Actinospica durhamensis* sp. nov., isolated from a spruce forest soil

**DOI:** 10.1007/s10482-015-0496-1

**Published:** 2015-05-31

**Authors:** Patrycja Golinska, Tiago Domingues Zucchi, Leonardo Silva, Hanna Dahm, Michael Goodfellow

**Affiliations:** Department of Microbiology, Nicolaus Copernicus University, 87 100 Toruń, Poland; Laboratório de Microbiologia Ambiental, EMBRAPA Meio Ambiente, Rod SP 340-Km 127, 5, PO Box 69, Jaguariúna, 13820-000 Brazil; School of Biology, University of Newcastle, Newcastle upon Tyne, NE1 7RU UK

**Keywords:** Actinobacteria, Polyphasic taxonomy, *Actinospica durhamensis* sp. nov., Spruce forest soil

## Abstract

Seven acidophilic actinobacteria isolated from humus and mineral layers of a spruce forest soil were examined using a polyphasic approach. Chemotaxonomic properties of the isolates were found to be consistent with their classification in the genus *Actinospica*. The strains formed a distinct phyletic line in the *Actinospica* 16S rRNA gene tree being most closely related to *Actinospica robiniae* DSM 44927^T^ (98.7–99.3 % similarity). DNA:DNA relatedness between isolate CSCA57^T^ and the type strain of *A. robiniae* was found to be low at 40.8 (±6.6) %. The isolates were shown to have many phenotypic properties in common and were distinguished readily from the type strains of *Actinospica acidiphila* and *A. robiniae* using a range of phenotypic features. On the basis of these data the seven isolates were considered to represent a new species for which the name *Actinospica durhamensis* sp. nov. is proposed. The type strain of the species is CSCA 57^T^ (=DSM 46820^T^ = NCIMB 14953^T^).

**Electronic supplementary material:**

The online version of this article (doi:10.1007/s10482-015-0496-1) contains supplementary material, which is available to authorized users.

## Introduction

The actinobacterial genus *Actinospica* is the sole representative of the family *Actinospicaceae* of the order *Catenulisporales* (Donadio et al. 2010a, b). The genus was proposed by Cavaletti et al. (2006) to accommodate two strains isolated from a soil sample collected from a wooded area in Gerenzano, Italy, namely *Actinospica acidiphila* GE134766^T^ and *Actinospica robiniae* GE134769^T^. The strains were found to be obligate acidophiles (pH range 4.2–6.2) that shared a combination of chemotaxonomic and morphological properties, notably the presence of 3-hydroxydiaminopimelic acid in whole-organism hydrolysates, and were distinguished using a battery of cultural and chemical markers (Cavaletti et al. 2006; Donadio et al. 2010c).

In a continuation of our bioprospecting studies on acidophilic and acidotolerant filamentous actinobacteria isolated from a spruce forest soil (Golinska et al. 2013a, b, c) many slow-growing obligate acidophilic strains were found to have colonial properties consistent with their assignment to the genus *Actinospica*. A polyphasic taxonomic study was carried out on seven of these isolates in order to establish their taxonomic status. The resultant data showed that the isolates form a new *Actinospica* species for which we propose the name *Actinospica durhamensis* sp. nov.

## Materials and methods

### Organisms, maintenance and biomass preparation

The organisms, strains HGG19, HSCA45, A1GG17, A1GG29, A1SCA6, CGG1, CSCA57^T^, were isolated from the humus (H), A1 and C mineral layers of a spruce soil at Hamsterley Forest, County Durham; the site and the dilution plate procedures used to isolate the strains have been described elsewhere (Golinska et al. 2013a, c). They were isolated from acidified starch-casein plates (Kűster and Williams 1964) using either agar (SCA) or gellan gum (GG) as gelling agents. The isolates were maintained on acidified (pH 5.5) yeast extract-malt extract ISP medium 2 (Shirling and Gottlieb 1966) plates at 4 °C and as hyphal fragments in glycerol (20 % v/v) at −80 °C, as were *A. acidiphila* DSM 44926^T^ and *A. robiniae* DSM 44927^T^.

Biomass for the chemotaxonomic and molecular systematic studies was prepared by growing the isolates in shake flasks of acidified ISP 2 broth (pH 5.5) at 150 revolutions per minute for 3 weeks at 28 °C. Cells were harvested by centrifugation and washed twice in distilled water; biomass for the chemotaxonomic analyses was freeze-dried and that for the molecular work stored at −20 °C. Cells for the phenotypic tests was washed ten times with distilled water then diluted to give a turbidity of 5 on the McFarland scale and used to inoculate the test media.

### Phylogenetic analyses

Extraction of genomic DNA, PCR-mediated amplification of the 16S rRNA genes of the isolates and direct sequencing of the purified PCR products were carried out as described by Golinska et al. (2013a, c). The closest phylogenetic neighbours based on 16S rRNA gene similarities were found using the EzTaxon server (http://eztaxon-e.ezbiocloud.net/: Kim et al. 2012). The resultant 16S rRNA gene sequences were aligned with corresponding sequences of the type strains of *A. acidiphila* and *A. robiniae* and those of *Catenulispora* species, using ClustalW. Phylogenetic analyses were carried out using the MEGA5 (Tamura et al. 2011) and PHYML (Guindon and Gascuel 2003) software packages. Phylogenetic trees were inferred by using the maximum-likelihood (Felsenstein 1981), maximum-parsimony (Fitch 1971) and neighbour-joining (Saitou and Nei 1987) tree-making algorithms, and evolutionary distances generated using the distance model described by Jukes and Cantor (1969). The tree topologies were evaluated by a bootstrap analysis (Felsenstein 1985) of the neighbour-joining data based on 1000 resamplings using MEGA5 software. The root position of the unrooted trees were estimated using the 16S rRNA gene sequence of *Bifidobacterium bifidum* NBRC 14252^T^ (GenBank accession number NR 113873).

### DNA:DNA relatedness

Reciprocal DNA:DNA pairing assays were carried out between isolate CSCA 57^T^ and *A. robiniae* DSM 44927^T^ at the DSMZ (Braunschweig, Germany). Cells were disrupted by using a Constant System TS 0.75 KW machine (IUL Instruments, Germany) and the crude DNA lysates purified by chromatography on hydroxyapatite after Cashion et al. (1977). DNA–DNA hybridization was carried out as described by De Ley et al. (1970) using the modifications described by Huss et al. (1983) and a model Cary 100 Bio UV/VIS-spectrophotometer equipped with a Peltier-thermostatted 6 × 6 multicell changer and a temperature controller with an in situ temperature probe (Varian) at 70 °C.

### Chemotaxonomy

Standard chromatographic procedures were used to determine the isoprenoid quinone (Collins 1985), polar lipid (Minnikin et al. 1984) and whole-organism sugar (Hasegawa et al. 1983), composition of the seven isolates, using the type strain of *A. robiniae* DSM 44927^T^ as control. Cellular fatty acids of isolate CSCA57^T^ were extracted, methylated and determined by gas chromatography (Hewlett Packard instrument 6890) and analysed using the standard Sherlock Microbial Identification (MIDI) system, version 5 (Sasser 1990). The isomers of diaminopimelic acid of two representative strains, namely isolates A1SCA17 and CSCA57^T^, were determined by the identification service of the DSMZ (Braunschweig, Germany), as described by Schumann (2011). The G+C mol% of the DNA of strain CSCA57^T^ was determined following the procedure described by Gonzalez and Saiz-Jimenez (2002).

### Cultural and morphological properties

All of the isolates were examined for cultural and morphological properties following growth on acidified tryptone–yeast extract, yeast extract–malt extract, oatmeal, inorganic salts–starch, glycerol–asparagine, peptone–yeast extract–iron and tyrosine agars; ISP media 1–7, respectively (Shirling and Gottlieb 1966).

### Phenotypic tests

A broad range of phenotypic tests were carried out on the isolates and the type strains of *A. acidiphila* and *A. robiniae* using acidified yeast carbon base (Sigma) without amino acids and acidified yeast nitrogen base (Sigma) media for the nitrogen and carbon utilization tests, respectively. The isolates were also examined for their ability to degrade a range of organic compounds, as described by Williams et al. (1983) and to grow at various temperatures (4, 10, 15, 20, 25, 30, 35 and 40 °C), pH values (4.0, 4.5, 5.0, 5.5, 6.0, 6.5, 7.0 and 7.5) and sodium chloride concentrations (1, 3, 5, 7 and 10 %, w/v) using acidified ISP2 agar (Shirling and Gottlieb 1966); apart from the temperature tests all of the media were incubated at 28 °C for 3 weeks.

## Results and discussion

Relatively little attention has been paid to acidophilic filamentous actinobacteria even though they were discovered many years ago (Jensen 1928). It is now known that these organisms are common in acidic habitats, notably coniferous soils (Williams et al. 1971; Khan and Williams 1975; Goodfellow and Dawson 1978; Goodfellow and Simpson 1987) and are considered to have a role in the turnover of organic matter (Goodfellow and Williams 1983; Williams et al. 1984). It is important to clarify the taxonomy of acidophilic filamentous actinobacteria, not least because they are a source of antifungal agents (Williams and Khan 1974) and acid stable enzymes (Williams and Flowers 1978; Williams and Robinson 1981).

### Chemotaxonomic, cultural and phenotypic properties

All of the strains isolated from the H, A1 and C horizons of the Hamsterley Forest spruce soil were found to have menaquinone, polar lipid and whole-cell sugar patterns as well as phenotypic properties consistent with their classification in the genus *Actinospica* (Cavaletti et al. 2006; Donadio et al. 2010c). The isolates were shown to be aerobic, Gram-positive, non-acid-alcohol fast, nonmotile actinobacteria that grew from pH 4.0–6.0, produced extensively branched substrate mycelia, whole-organism hydrolysates that contained arabinose, galactose, mannose and rhamnose (trace), contained di, tetra-, hexa- and octa-hydrogenated menaquinones in the ratio 5, 22, 40 and 30 %, respectively, and major amounts of diphosphatidylglycerol, phosphatidylethanolamine (taxonomically significant component) and phosphatidylglycerol (phospholipid pattern 2 sensu Lechevalier et al. 1977; Online supplementary Fig. 1). The whole-organism hydrolysates of isolates A1SCA17 and CSCA57^T^ were found to contain 2, 6-diamino-3-hydroxy and *meso*-diaminopimelic acids in a ratio 1:2, respectively. The fatty acid profile of isolate CSCA57^T^ was shown to contain major proportions (>10 %) of *iso*-C_15:0_, (22.2 %), *anteiso*-C_15:0_ (14.2 %), *iso*-C_16:0_ (27.7 %) and *anteiso*-C_17:0_ (16.7 %), minor proportions (>1.1 %) of *iso*-C14 (1.65 %), C_16:0_ (3.5 %), *anteiso*-C_17:1_ω9c (1.7 %), *iso*-C_17:0_ (3.9 %), C_17:1_ω8c (1.1 %), C_17:0_ (1.1 %) and summed feature *iso*-C_17:1_ω9c (2.9 %) and trace amounts (<0.7 %) of a few components. This isolate was also found to have a DNA G+C ratio of 68.0 mol% and to grow to varying degrees on most of the ISP media producing substrate mycelial pigments that distinguished them from the type strains of *A. acidiphila* and *A. robiniae* (Table 1). None of the isolates formed aerial hyphae on the ISP media.

**Table 1:** Growth and cultural characteristics of the isolates and the type strains of *A. acidiphila* and *A. robiniae* on acidified ISP media after incubation for 4 weeks at 28 °C

Isolates	A1GG17, A1GG29, A1SCA6, CGG1, CSCA57^T^, HGG19, HSCA45		*A. acidiphila* DSM 449226^T^ (GE 134766^T^)			*A. robiniae* DSM 44927^T^ (GE 134769^T^)		
	Growth	Colour of substrate mycelia	Growth	Colour of: aerial spore mass	Substrate mycelium	Growth	Colour of: aerial spore mass	Substrate mycelium
Yeast extract–malt extract agar (ISP 2)	++++	Medium yellow	+++	White	Pale greenish yellow	+++	Whitish	Light greenish yellow
Oatmeal agar (ISP 3)	+++	Light yellowish brown	+++	White	Grayish yellow	+++	Whitish	Dark grayish yellow
Inorganic salts-starch agar (ISP 4)	+++	Greenish white	+++	Absent	White	+	Absent	White
Glycerol-asparagine agar (ISP 5)	++	White	++	White	Greenish white	+	Absent	White
Peptone-yeast extract iron agar (ISP 6)	+	Medium yellow	+	White	White	–	Absent	Absent
Tyrosine agar (ISP 7)	**++**	White	+++	White	Greenish white	++	Absent	White

The isolates either did not grow or showed scant growth on acidified tryptone-yeast extract agar. None of the strains formed diffusible pigments or aerial spore mass

*++++* abundant, *+++* good, *++* poor, *+* scant growth, *–* no growth

### 16S rRNA gene sequencing and DNA:DNA relatedness studies

**Fig. 1:**
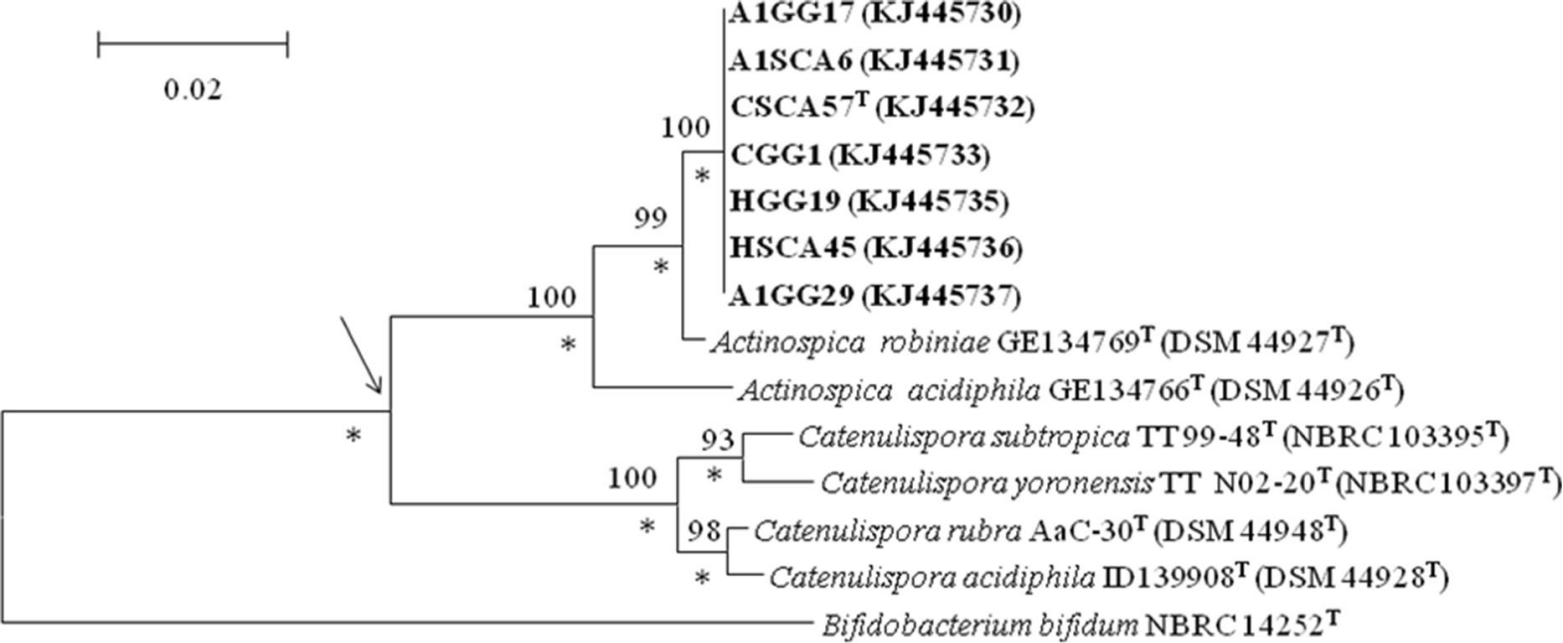
Neighbour-joining tree based on nearly complete 16S rRNA gene sequences (1399–1532 nucleotides) showing relationships between the isolates and between them and the type strains of *Actinospica* species*. Asterisks* indicate branches of the tree that were also found using the maximum-likelihood and maximum-parsimony tree-making algorithms. *Numbers at the nodes* indicate the percentage bootstrap values based on 1000 re-sampled datasets, only values above 50 % are given. *T* type strain. *Bar* 0.02 substitutions per nucleotide position. The root position of the tree was determined by using *Bifidobacterium bifidum* NBRC 14252T as outgroup

Near complete 16S rRNA gene sequences of isolates A1GG17, A1GG29, A1SCA6, CGG1, CSCA57^T^, HGG19 and HSCA45 (GenBank accession numbers; KJ 445730, KJ 445737, KJ 445731, KJ 445733, KJ 445732, KJ 445735 and KJ 445736, respectively) were generated. The isolates were found to have identical or almost identical 16S rRNA gene sequences (99.9–100 % nucleotide [nt] similarity) and were shown to form a branch in the *Actinospica* gene tree that was supported by all of the tree-making algorithms and by a 100 % bootstrap value (Fig. 1). In turn, the isolates were found to be most closely related to the type strain of *A. robiniae* DSM 44927^T^, a relationship that was underpinned by a 100 % bootstrap value and by all of the tree-making methods. The isolates shared 16S rRNA gene similarities with *A. robiniae* DSM 44927^T^ that fell within the range 98.7 and 99.3 %, values equivalent to 10–18 nt differences at 1399–1421 locations. Corresponding 16S rRNA gene sequence similarities between the isolates and the type strain of *A. acidiphila* lay within the range 96.6–97.2 %, values that corresponded to between 40 and 49 nt differences at between 1397 and 1421 locations. The *Actinospica* 16S rRNA gene clade was distinguished readily from a second clade accommodating the type strains of *Catenulispora* species (Fig. 1). The isolates were also shown to exhibit the pattern of 16S rRNA gene signatures characteristic of members of the family *Actinospicaceae* (Donadio et al. 2010b).

The DNA:DNA similarity value between isolate CSCA57^T^ and *A. robiniae* DSM 44927^T^ was shown to be 40.8 (±6.6)  %, a result that is well below the 70 % cut-off point recommended for assigning bacterial strains to the same genomic species (Wayne et al. 1987).

### Phenotypic tests

The isolates had many phenotypic features in common, some of which were shown to distinguish them from the type strains of *A. acidiphila* and *A. robiniae* (Table 2). The isolates, unlike *A. robiniae* DSM 44927^T^, their nearest phylogenetic neighbour, grew on d-trehalose as a sole carbon source, on l-alanine, l-*iso*leucine, l-phenylalamine and l-valine as sole nitrogen sources and in the presence of 1 %, w/v sodium chloride. Conversely, the *A. robiniae* strain, unlike the isolates grew on d-lactose and d-raffinose as sole carbon sources. Some, but not all, of the isolates grew at pH 6.5 and used l-histidine as a sole nitrogen source. It can also be seen from Table 2 that the isolates were found to have sugar and polar lipid patterns that distinguish them from the *A. acidiphila* and *A. robiniae* strains. The source of the isolates and features that distinguish them are shown in Table 3.

**Table 2:** Phenotypic properties that distinguish the isolates from the type strains of *A. acidiphila* and *A. robiniae*

Characteristic	Isolates A1GG17, A1GG29, A1SCA6, CGG1, CSCA57^T^, HGG19, HSCA45	*A. acidiphila* DSM 44926^T^ (GE 134766^T^)	*A. robiniae* DSM 44927^T^ (GE 134769^T^)
Degradation of Tween 20	**–**	**–**	**+**
Growth on sole carbon sources (1 %, w/v)			
d-Lactose	–	–	+
d-Raffinose	–	+	+
d-Trehalose	+	+	–
Growth on sole nitrogen sources (0.1 %, w/v)			
Acetamide	+	+	–
l-Alanine	+	+	–
l-Isoleucine	+	+	–
l-Phenylalanine	+	–	–
l-Valine	+	+	–
Growth at 15 °C	+	–	–
pH 4.0	+	–	–
Growth in presence of 1 %, w/v NaCl	+	+	–
Chemotaxonomy			
Diagnostic sugars in whole-organism hydrolysates	Ara, gal, man, rham (trace)	Ara, man, rham, xyl	Gal, man, rham
Predominantphospholipids	DPG, PE, PG, PI	DPG, PE, methyl-PE, PI	DPG, PE, methyl PE, PI
GC content of DNA (mol%)	68.0	69.2	70.8

The chemotaxonomic data on the type strains of *A. acidiphila* and *A. robiniae* was taken from Cavaletti et al. (2006)

*+* positive, *−* negative, *Ara* arabinose, *gal* galactose, *man* mannose, *rham* rhamnose, *xyl* xylose, *DPG* diphosphatidylglycerol, *PE* phosphatidylethanolamine, *methyl-PE* methyl phophatidylethanolamine, *PG* phosphatidylglycerol, *PI* phophatidylinositol

**Table 3:** Properties that distinguish between isolates

Characteristics	HGG19	HSCA45	A1GG17	A1GG29	A1SCA6	CGG1	CSCA57^T^
Source	Humus horizon H	Humus horizon H	Mineral horizon A1	Mineral horizon A1	Mineral horizon A1	Mineral horizon C	Mineral horizon C
Growth at pH 6.5	−	−	+	−	+	−	−
Growth on l-histidine as a sole nitrogen source	+	−	+	+	−	−	+
H_2_S production	+	+	+	+	−	+	+

*+* positive, *−* negative

## Conclusion

The genotypic and phenotypic profiles of the isolates show that they form a well-circumscribed taxon within the genus *Actinospica*. It is, therefore, proposed that they represent a novel species, *Actinospica durhamensis* sp. nov.

### Description of *Actinospica durhamensis* sp. nov.

*Actinospica durhamensis* (dur. ham. en’ sis. N.L. fem. adj. *durhamensis*, belonging to Durham, a county in the North East of England, the source of the isolates).

Aerobic, Gram-positive, non-acid-alcohol-fast, acidophilic actinobacteria which form an extensively branched substrate mycelium, but do not form aerial hyphae on ISP media. Strains grow at 15–33 °C, optimally ~28 °C, from pH 4.0 to 6.0, optimally at pH 5.5 and in the presence of 1 % w/v, but not 3 %, w/v sodium chloride. Tweens 40 and 60 are metabolised, but not adenine, casein, chitin, elastin, gelatin, guanine, hypoxanthine, starch, Tweens 20 and 80, tyrosine, uric acid, xanthine or xylan. l-arabinose, d-cellobiose, dextran, d-fructose, d-galactose, d-glucosamine, d-glucose, glycogen, d-melibiose, β-methyl-α-glucoside, l-rhamnose and d-ribose are used as sole carbon sources for energy and growth, but not adonitol, amygdalin, d- or l-arabitol, *meso*-erythritol, d-glucuronic acid, glycerol, *meso*-inositol, inulin, d-lactose, d-mannitol, d-melezitose, α-methyl-β-glucoside, d-raffinose, d-salicin, d-sucrose or xylitol (all at 1 %, w/v). Acetate, adipate, benzoate, butyrate, citrate, fumarate, hippurate, oxalate, propionate, pyruvate and succinate (sodium salts) or *p*-hydroxybenzoic acid (all at 0.1 %, w/v) are not used as sole sources of carbon. l-arginine, l-asparagine, l-aspartic acid, l-cysteine, l-glutamic acid, l-hydroxyproline, l-methionine, l-serine and l-threonine are used as a sole nitrogen sources (all at 0.1 %, w/v). Additional phenotypic properties are cited in the text and in Tables 1 and 2. The major fatty acids are *iso*-C_15:0_, *anteiso*-C_15:0_, *iso*-C_16:0_ and *anteiso*-C_17:0_. The G+C content of the DNA of the type strain is 68.0 mol%. Additional chemotaxonomic properties are given in the text and in Table 2.

The species contains the type strain CSCA57^T^ (=DSM 46820^T^ = NCIMB 14953^T^) and isolates HGG19, HSCA45, A1SCA6, A1GG29, A1GG17, CGG1, all of which were isolated from spruce soil from Hamsterley Forest, County Durham, England. The Genbank accession number of the 16S rRNA gene sequence of strain CSCA57^T^ is KJ445732.

## Electronic supplementary material

Supplementary Fig. 1 Two dimensional thin-layer chromatography of polar lipids of isolate CSCA57^T^ stained with molybdenum blue (Sigma). Chloroform : methanol : water (32.5 : 12.5 : 2.0 v/v) were used in the first direction and chloroform : acetic acid : methanol : water (40 : 7.5 : 6 : 2 v/v) in the second direction. DPG, diphosphatidylglycerol; PE, phosphatidylethanolamine; PI, phosphatidylinositol; PIMS, phosphatidylinositol mannosides (DOC 805 kb)
