# Supplementary figures and images for: Actinospica durhamensis sp. nov., isolated from a spruce forest soil
Source: Antonie Van Leeuwenhoek. 2015 May 31;108(2):435–42. doi: 10.1007/s10482-015-0496-1 (PMC4491342; doi:10.1007/s10482-015-0496-1)

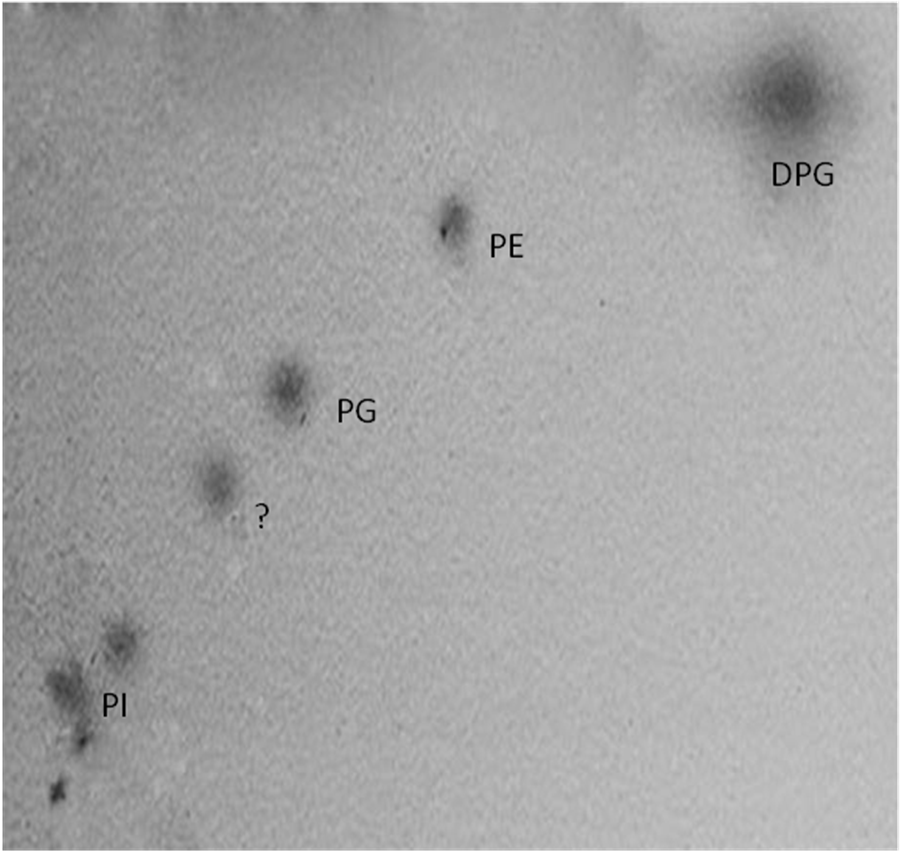

Supplement: Supplementary file 1 — Supplementary Fig. 1 Two dimensional thin-layer chromatography of polar lipids of isolate CSCA57T stained with molybdenum blue (Sigma). Chloroform : methanol : water (32.5 : 12.5 : 2.0 v/v) were used in the first direction and chloroform : acetic acid : methanol : water (40 : 7.5 : 6 : 2 v/v) in the second direction. DPG, diphosphatidylglycerol; PE, phosphatidylethanolamine; PI, phosphatidylinositol; PIMS, phosphatidylinositol mannosides (DOC 805 kb) [file 10482_2015_496_MOESM1_ESM.doc]
